# *War and Cancer*: Exploring Patient Narratives in a Randomized Integrative Oncology Study

**DOI:** 10.1002/pon.70172

**Published:** 2025-05-06

**Authors:** Eran Ben‐Arye, Orit Gressel, Yael Keshet, Vika Zaritsky, Sameer Kassem, Yakir Segev, Noah Samuels

**Affiliations:** ^1^ Integrative Oncology Program The Oncology Service; Lin, Carmel, and Zebulun Medical Centers Clalit Health Services Haifa Israel; ^2^ Ruth and Bruch Rappaport Faculty of Medicine Technion‐Israel Institute of Technology Haifa Israel; ^3^ Department of Sociology Western Galilee Academic College Acre Israel; ^4^ Department of Internal Medicine Lady Davis Carmel Medical Center Haifa Israel; ^5^ Unit of Gynecologic Oncology Department of Obstetrics and Gynecology Carmel Medical Center Haifa Israel; ^6^ Center for Integrative Complementary Medicine Shaare Zedek Medical Center Hebrew University of Jerusalem Jerusalem Israel

**Keywords:** cancer, integrative medicine, integrative oncology, pain, supportive and palliative care, war

## Abstract

**Study Objective:**

The current war in Israel has affected quality of life (QoL)‐related concerns among patients with cancer. Integrative oncology (IO) provides complementary medicine within supportive and palliative care. The study examined an IO program in northern Israel for cancer and war‐related concerns.

**Methods:**

This qualitative study, nested within a prospective randomized controlled trial, examined patients undergoing oncology and palliative care expressing cancer and war‐related QoL concerns referred by oncology healthcare providers to an IO program. Patients were randomized to manual‐relaxation with (Group A) or without (Group B) acupuncture. Narratives were recorded in MYCaW (Measure Yourself Concerns and Well‐being) questionnaires immediately and after 24 h. Narrative excerpts were qualitatively analyzed using ATLAS.Ti software for systematic coding.

**Results:**

Patient narratives (Group A, 67; Group B, 58) focused primarily on themes which included a sense of calmness and reduced emotional distress (e.g., stress, anxiety, worries, nervousness), more prominently in Group B (manual‐relaxation only; 55/56 immediately/after 24 h); and reduced pain severity, more so in Group A (with acupuncture; 19/24). Most excerpts described improved QoL in both groups (142/147, 97%) immediately after IO treatments, less so at 24 h (103/135).

**Conclusions:**

Narratives of patients facing cancer‐ and war‐related QoL challenges due to the ongoing war in Israel describe a beneficial impact of IO treatments, both immediately and after 24 h. Manual‐relaxation without acupuncture was more likely to improve emotional concerns, with pain relief more apparent with the addition of acupuncture. Further qualitative and quantitative research is needed to explore these findings.

## 1 Background

Integrative oncology (IO) programs provide evidence‐based complementary medicine therapies within supportive and palliative care services in leading cancer centers across the globe. The vast amount of research on the safety and effectiveness of these programs in addressing QoL‐related concerns among these patients has led to the publication of clinical practice guidelines, such as those being co‐published by the Society for Integrative Oncology (SIO) and American Society of Clinical Oncology (ASCO) on IO care for cancer‐related pain [1], anxiety and depression [2], and fatigue [3]. The ongoing research being published in the palliative care and psycho‐oncology literature has been focused primarily on the impact of IO on patient‐centered care, examining quality of life (QoL)‐related concerns and suffering.

A number of factors may contribute to QoL‐related concerns among patients and informal caregivers, in addition to their own experiences and coping abilities with advanced tumor stage and progression; adverse effects of oncology treatments; as well as a wide range of bio‐psycho‐social, cultural, and spiritual challenges which are unrelated to the cancer diagnosis. Finally, patients and informal caregivers may face more broad or extensive groups of challenges, including those on a national or even global scale. This was seen during the recent COVID‐19 pandemic, which was shown to significantly impair the wellbeing of older patients with cancer [4].

In contrast to the large body of research on the impact of IO programs on patients with cancer, little has been published on their impact on wellbeing during national and global crises, though some research was done in hospital‐based COVID‐19 settings [5]. At the same time, researchers have examined the impact of non‐oncology integrative medicine practices on a national level, such as a study examining outcomes from an acupuncture intervention provided to Middle Eastern refugees in the United Kingdom [6]. The present study qualitatively explored the narratives of patients with cancer participating in an IO program, addressing QoL‐related concerns and wellbeing related to both the cancer and its treatment and to the current war in Israel.

## 2 Materials and Methods

### 2.1 Study Design and Population

This study methodology was qualitative, nested within a prospective randomized study which took place at the Oncology Service of Clalit Healthcare Services in Lin, Carmel, and Zebulon Medical Centers in northern Israel [7]. Patient recruitment took place from August 2024 to January 2025, in the midst of the currently ongoing war in both southern and northern Israel. Patients aged ≥ 18 years undergoing oncology treatment, whether in an out‐patient (oncology daycare service) or in‐patient (gynecological oncology and internal medicine departments) setting were eligible for study inclusion.

## 3 Referral to IO Treatments

Patients were referred to the IO consultation by one of their health care providers, including oncology and palliative nurses, physicians (oncologists, gynecological oncologists, and internal physicians) and psycho‐oncologists. Patient referrals required at least one cancer‐related QoL indication (e.g., adverse effects of the oncology treatment) and a concurrent war‐related QoL concern (e.g., emotional distress, difficulty in coping). Patients referred to the IO program were first seen by an integrative physician, a medical doctor dually trained in IO and supportive cancer care. The IO consultation entailed an in‐depth explanation of the study protocol, following which the patient was ask to sign the study informed consent form. The integrative physician and patient then identified the leading QoL‐related concerns, co‐defining treatment goals. The patient was then scheduled for weekly IO treatments, for a duration of 3 weeks.

### 3.1 Allocation to Study Arms

Study patients were randomly allocated to one of two IO treatment groups, using block randomization of 8 with an allocation ratio of 1:1 (Research Randomizer tool, https://www.randomizer.org/) in each of the participating medical centers and departments: Group A, manual‐relaxation treatment, combined with acupuncture; or Group B, manual‐relaxation treatment only (without acupuncture).

Weekly IO treatments were tailored in accordance with the treatment goals which had been co‐defined with the patient, with each session lasting approximately 30 min. Manual‐relaxation treatments were provided by an IO practitioner trained in touch (e.g., acupressure, reflexology) and relaxation modalities (e.g., breathing, relaxation and guided imagery). Acupuncture treatments (Group A only) was administered by IO practitioners who were dually trained in acupuncture, acupressure, and relaxation modalities. All IO practitioners were required to undergo a 280‐h IO training program, with at least 5 years of work experience in the IO program at one of the three study centers, using semi‐structured IO protocols for the treatment of pain and anxiety.

### 3.2 Narrative Assessment and Analysis

Assessment of patients' response to the IO treatment program regarding QoL‐related concerns was conducted at baseline; immediately following treatment; at 24–48 h post‐treatment; and 3 weeks later. Patient narratives were reported in follow‐up MYCaW questionnaires [8], which ask patients to respond in writing to two 2 open‐ended questions regarding other therapeutic options which they felt had been helpful; and “what has been the most important aspect for you?” with respect to the IO treatments. Short narratives in the second question were assessed both qualitatively as well as quantitively, using ATLAS.ti Scientific Software (V.8) for analysis, enabling systematic coding. A qualitative content analysis was conducted using a conventional content analysis, thereby avoiding the need for pre‐established categories for coding [9, 10]. Short narratives were read one line at a time, assigning codes to the sections of each narrative accordingly. Quotes shared by each code were collated, followed by sorting the codes into categories based on their relationship with each other; then grouped into meaningful clusters with emerging themes. The number of quotes (i.e., “narrative excerpts”, where a single narrative may include several excerpts) were then quantified within the categories, followed by a comparative analysis of the results.

### 3.3 Ethical Considerations

The Ethics Review Board (Helsinki Committee) at the Carmel Medical Center in Haifa, Israel approved the study protocol (CMC‐24–0046), and it has been registered at ClinicalTrials.gov (NCT06604455). Participation in the study was voluntary, with no incentive offered such as payment or the like.

## 4 Results

### 4.1 Description of the Study Group

A total of 125 patients were recruited, with 67 allocated to Group A (manual‐relaxation treatment with acupuncture), and 58 to Group B (without acupuncture). The overall cohort was predominantly female (*n* = 70, 56%), with a median age of 65 years and with a reported average‐to‐high income (77, 62%) and higher education (85, 68%). The majority of patients self‐identified as Jewish (94, 75%), primarily secular (80, 64%); attended the IO consultation accompanied by an informal caregiver (spouse: 75, 60%; child: 23, 18%); and reported prior experience with complementary medicine therapies (96, 77%). Most were currently undergoing active oncology treatment, in either an adjuvant (18, 14%), neo‐adjuvant (16, 13%) or palliative care setting (51, 41%). The most predominant cancer diagnoses were breast (51), gynecologic (39), and gastro‐intestinal tumors (19), with similar distribution between the study groups.

### 4.2 Post‐Treatment Narratives: Immediate and 24 h After Treatment

An analysis of patient MYCaW narratives immediately after the IO treatments identified 61 patients referring to calmness/relaxation (with 37 referring to other emotional outcomes). Patients also related to pain (16 patients), neuropathy (12), fatigue/drowsiness (10), nausea (3), and other Qol‐related concerns. In both immediate and 24‐h post‐treatment groups, the majority of narrative excerpts (142/147, 97%) suggested improved QoL attributed to the IO treatments, though less (though still positive) at 24 h (103/135, 76%). And while 61 patients referred to a “calming” effect immediately post‐treatment, only 29 reported this at the 24‐h assessment. In addition, while 16 patients reported pain relief immediately after treatment, and 22 at 24 h, the beneficial effect on pain decreased between the two assessments from 14/16 (87.5%) to 14/22 (64%).

### 4.3 Calming Effect and Other Emotional Concerns

In both study groups (A and B), patients reported a similarly enhanced feeling of calm, referring to a relaxed, pleasant, peaceful; some even a state of release, or hypnosis (“*I was almost in a hypnotic state listening to you*”); and some comparing the feeling to that of a drug‐like effect (“*I feel entirely calm… as if I took a sedative*…”). While this feeling was expressed in 61/147 narrative excerpts (41%) immediately after the IO treatment, it persisted in only 29/135 (21%) of the 24‐h narratives.

Yesterday, after and during the touch therapy, I had a peaceful floating feeling, something that doesn't happen to me in real life.

(73‐year‐old woman undergoing oncology treatment for advanced ovarian cancer)

Other emotional‐related concerns were expressed in these narratives, including hope, security, and optimism. At the same time, IO treatments were associated with a sense of “strengthening”, “energizing”, “refreshing” and “elation”, in addition to providing relief from worries, anxiety and tension. Emotional‐related concerns were dependent, most significantly in Group B (manual‐relaxation only), on the patient's rapport with the IO practitioner.

You are a ray of light in the darkness here…very physically pleasant…all this attention, this support, shows that you care…with empathy in this entire cold system….

(64‐year‐old woman with advanced endometrial carcinoma, hospitalized for cancer‐related fatigue)

In most of the narratives, the war itself was rarely mentioned directly in association with calming/emotional‐related keywords. However, it was present, if only indirectly.

Breathing continues to be good now…it's okay now. I don't feel the tightness in my diaphragm anymore. We're already used to the missiles, and there were already a few sirens this morning…there is a lot of fear about other things…we are under siege, because of the current situation ….

(72‐year‐old woman undergoing neo‐adjuvant chemotherapy for breast cancer)

Several patients described the calming effect within both a physical and spiritual context (“*You gave me peace, and a chance to connect with my body and mind*”). This mind‐body relatedness was also described in reference to pain.

It is amazing how connected the body and mind are… how much inner pain I began to feel when I started to understand*….*

(65‐year‐old woman with advanced lung cancer)

However, associating mind and body was not always described as a peaceful experience.

Experiences like this give me the strength to understand that body and soul are one… I need to find that something that reaches my soul… I am learning from this process what needs to be changed, what needs to be fixed… I am a very closed person, trying to reach places which are deep…to understand what is needed in life….

(42‐year‐old male with metastatic rectal adenocarcinoma)

### 4.4 Narratives of Pain

Pain‐related narrative excerpts were more prevalent in Group A (manual‐relaxation treatment, with acupuncture) than in Group B (without acupuncture), with more patients in the former describing an improvement following IO treatment (Group A, 19/24; vs. 4/6 in Group B). Pain‐related symptoms were used to describe headache, back, abdominal, legs (including improved ability to walk) and generalized pain.

Acupuncture treatment is a time when there is no pain anywhere in the body… no place burns or pulsates….

(62‐year‐old female with suspected breast cancer recurrence)

In some of the narratives, patients associated the reduction in pain with reduced stress and better sleep, as well as daily functioning.

I'm in less pain… I can start the day anew…I slept last night like I haven't slept for a while…now, I can start the day….

(41‐year‐old Arab‐speaking woman with metastatic breast cancer)

When compared to the impact of IO treatments on emotional‐related concerns, some patients reported less of an improvement for pain at 24 h post‐treatment. This was attributed by the patients to a reduced effect of IO treatments with time (within the first 24 h, more after that); the tendency of pain to recur; additional causes of pain, such as acupuncture, neuropathy; or to overwhelming concerns about the ongoing war in Israel.

Right after the treatment I really felt good, which lasted until the evening. There was fatigue, but no terrible headaches. Now the symptoms of the chemo are returning, slowly…slowly…however, I didn't need to do my breathing exercises, even though it had been a long and busy day (referring to a recent missile attack)….

(56‐year‐old woman undergoing neo‐adjuvant chemotherapy for breast cancer)

## 5 Discussion

The present qualitative study explored narratives of patients who, in addition to their cancer diagnosis and treatment, are confronted with the additional trauma and stresses of the ongoing war in Israel. The majority of these patients were currently undergoing oncology and/or palliative treatments which significantly impaired their QoL and wellbeing, as a result of complications of advanced cancer and the toxicities of their anti‐cancer treatment regimens. The study focused on patient MYCaW narratives, addressing the impact of the IO program immediately after treatments and 24 h later. The study is imbedded within a randomized controlled trial, comparing a manual‐relaxation regimen, with acupuncture or on its own. Qualitative analysis of short patient narratives in both study groups suggests a significant calming effect of the intervention. This included a broad spectrum of emotional‐related concerns which improved significantly for patients, most significantly immediately after treatment, less pronounced at the 24‐h assessment. And while narratives on the “calming” effect and relief of emotional‐related concerns are very rich and descriptive, they were focused almost exclusively on the oncology setting, and only rarely on the current and ongoing war in the country.

The published qualitative research examining the effects of war on patients with cancer has been focused primarily on the limited accessibility to cancer care in areas across the globe, such as the Kurdish region of Iraq [11], the Nagorno‐Karabakh region in the South Caucasus [12], and Northwest Syria [13]. Little of this research examined the perception of patients with cancer on how the conflict has affected their cancer journey, which in and of itself is often called a “battle” or “war on cancer” [14].

### 5.1 Clinical Implications

In the present study, it is possible that the IO interventions were able to provide a calming effect for both the cancer and the stress of the ongoing war, each from its own perspective. In addition to addressing cancer‐related concerns, the IO setting may have created a type of “safe place” in which the patient was able, if only for the duration of the treatment, to forget the conflict taking place outside. This may not necessarily reflect specific effects generated by the IO treatments, but rather non‐specific effects resulting from the creation of rapport and bonding during the interaction between the patient and the IO practitioner. It is therefore of interest that while patients in both groups received manual‐relaxation treatments, with or without acupuncture, the calming effect was more evident in Group B, which received only the manual‐relaxation treatment (without acupuncture).

In contrast, pain relief was more pronounced in Group A, in which patients received acupuncture in addition to manual‐relaxation treatments. While the analysis performed in this study was qualitative, it is possible that there was an additive effect provided by acupuncture for pain relief, suggesting a more specific (rather than non‐specific) effect. The analgesic effect of the IO intervention (in both groups) was most significant in patient narratives immediately following the IO treatment, fading to some extent during the 24 h that followed. If this is indeed reflective of the true numbers, it needs to be better understood why the calming effect was greater with manual‐relaxation treatment alone, and pain reduction with the addition of acupuncture. A similar picture was reported in a study of patients with taxane‐induced peripheral neuropathy, who were randomized to acupuncture alone versus acupuncture combined with manual‐movement/mind‐body modalities [15]. Here, too, the acupuncture‐only group showed a greater improvement for “physical” concerns, such as numbness and tingling of the hands; and the multi‐modality group improved more significantly on emotional wellbeing scales. Further research is needed to examine whether the impact of acupuncture is indeed greater for physical symptoms such as pain; and mind‐body and manual‐relaxation treatments primarily for emotional‐related and calming effects.

### 5.2 Study Limitations

This study has a number of methodological limitations which need to be addressed in future research. Firstly, the qualitative methodology examined only short self‐reported patient narratives, without conducting in‐depth qualitative interviews that may have identified whether and to what extent the effects of the IO treatment addressed war‐related concerns. The findings of the narrative assessment may have also reflected both non‐specific (e.g., being taken care of; a safe and quiet setting) and specific (direct effects of IO treatments on QoL‐related concerns, such as pain). Finally, the generalizability of the study for other centers with diverse social‐cultural populations remains to be explored.

### 5.3 Conclusion

The narratives of patients faced with both cancer‐ and war‐related QoL challenges describe a beneficial effect from the IO treatment program, most significantly immediately after treatment and with the effect less pronounced after 24 h. Emotional‐related concerns and a sense of calmness were more significant in the narratives of patients treated with manual‐relaxation alone; and pain relief more predominant in those treated with the addition of acupuncture. Further research is needed to explore these findings, using both qualitative as well as quantitative outcome analysis, including physiological parameters.

## Author Contributions

E.B‐A., O.G., and V.Z. organized the trial and collected the data analyzed in this study. E.B‐A, O.G., V.Z., and S.K. planned the study. Y.K., E.B‐A., and O.G. carried out the analysis and wrote a draft manuscript. All authors participated in the revision of the manuscript.

## Ethics Statement

The Ethics Review Board (Helsinki Committee) at the Carmel Medical Center in Haifa, Israel approved the study protocol (CMC‐24–0046), and it has been registered at ClinicalTrials.gov (NCT06604455).

## Consent

The study participants provided written informed consent.

## Conflicts of Interest

All authors declare that they have received no support from any organization for the submitted work; have no financial relationships with any organizations that might have an interest in the submitted work during the previous 3 years; and have no other relationships or activities that could appear to have influenced the submitted work.

## Data Availability

The data that support the findings of this study are available on request from the corresponding author. The data are not publicly available due to privacy or ethical restrictions.
